# Age and sex differences in soluble ACE2 may give insights for COVID-19

**DOI:** 10.1186/s13054-020-02942-2

**Published:** 2020-05-14

**Authors:** Per Swärd, Andreas Edsfeldt, Anton Reepalu, Lars Jehpsson, Björn E. Rosengren, Magnus K. Karlsson

**Affiliations:** 1 Clinical and Molecular Osteoporosis Research Unit, Departments of Orthopedics and Clinical Sciences, Skåne University Hospital, Lund University, Malmö, Sweden; 2 Department of Cardiovascular Research-Translational Studies and Cardiology, Skåne University Hospital, Lund University, Malmö, Sweden; 3 Department of Translational Medicine and Clinical Infection Medicine, Skåne University Hospital, Lund University, Malmö, Sweden

To the editor:

mACE2 (membrane-bound angiotensin-converting enzyme 2) is central when developing severe COVID-19 (coronavirus disease 2019), because SARS-CoV-2 (severe acute respiratory syndrome coronavirus-2) attaches to the active surface domain of mACE2 when entering the host cell [1]. ADAM-17 (a disintegrin and metalloproteinase-17), during physiological conditions and in SARS-CoV infection, can cleave mACE2, resulting in shedding and soluble ACE2 (sACE2) [2, 3], a process also associated with ALI (acute lung injury) [1]. High mACE2 and/or high ADAM-17 activity may therefore facilitate SARS CoV-2 infection and severe COVID-19, and sACE2 may capture this risk, reflecting (i) high mACE2, (ii) high ADAM-17 activity, or (iii) both.

Severe COVID-19 is more common in adults than in children and in men than women [4]. This may be related to differences in mACE2 expression and/or age-related alterations in the RAS (renin-angiotensin system), associated with increased angiotensin II/ADAM-17 activity and increased mACE2 shedding [1, 2, 5]. The aim of this study was to evaluate sACE2 levels during growth and compare results by sex and age.

We analyzed sACE2 in serum collected at mean ages (SD) 9.9 (0.6), 11.7 (0.6), 14.8 (0.8), 18.8 (0.3), and 23.5 (0.7) years in individuals in the pediatric osteoporosis prevention (POP) study, a prospective study that investigates the effects of physical activity on musculoskeletal development through growth (Ethics Committee of Lund University, Sweden LU 2015/118) [6]. sACE2 was analyzed by Olink® Inflammation Cardiovascular II panel. Data is presented as Normalized Protein eXpression (NPX) values, which is an arbitrary unit on a log2 scale (Olink Proteomics AB, Uppsala, Sweden; http://www.olink.com). Seven outlier observations of sACE2 that deviated more than 3 SD from the gender-specific mean were removed from the analyses. Characteristics of subjects included in the analyses of the present study are presented in Table 1. Differences in sACE2 between male and female subjects were calculated using analysis of covariance, adjusted for age. The level of significance was set at *p* < 0.05, and analyses were performed using the SPSS statistical package (v26; SPSS Inc., Chicago, Ill).

**Table 1:** Subject background data and sACE2 levels in relation to age and sex

	**Baseline, age 7.7 (SD 0.6) years**		**Age 9.9 (SD 0.6) years**		**Age 11.7 (SD 0.6) years**	
	Boys (*n* = 191)	Girls (*n* = 158)	Boys (*n* = 92)	Girls (*n* = 80)	Boys (*n* = 88)	Girls (*n* = 67)
***Background data***						
Age, years (SD)	7.7 (0.6)	7.7 (0.6)	10.0 (0.6)	9.8 (0.6)	11.8 (0.6)	11.7 (0.6)
Height, cm (SD)	128.8 (6.5)	128.0 (7.0)	140.6 (6.8)	140.1 (7.3)	152.5 (8.0)	152.6 (10.0)
Weight, kg (SD)	27.7 (5.3)	27.3 (5.3)	34.3 (6.9)	34.4 (6.6)	43.4 (9.2)	43.8 (9.6)
BMI, kg/m^2^ (SD)	16.6 (2.3)	16.6 (2.4)	17.3 (2.6)	17.5 (2.7)	18.5 (2.9)	18.6 (3.3)
***Outcome***						
sACE2 (NPX)	N/A	N/A	3.3 (0.3)	3.3 (0.3)	3.3 (0.3)	3.3 (0.3)
	**Age 14.8 (SD 0.8) years**		**Age 18.8 (SD 0.3) years**		**Age 23.5 (SD 0.7) years**	
	Boys (*n* = 82)	Girls (*n* = 66)	Boys (*n* = 48)	Girls (*n* = 44)	Men (*n* = 75)	Women (*n* = 74)
***Background data***						
Age, years (SD)	14.9 (0.7)	14.7 (0.8)	18.8 (0.3)	18.8 (0.3)	23.5 (0.7)	23.5 (0.7)
Height, cm (SD)	173.2 (8.2)	165.7 (6.7)	181.8 (6.5)	168.5 (5.0)	180.6 (6.9)	168.6 (5.9)
Weight, kg (SD)	61.8 (13.2)	57.6 (11.0)	75.9 (11.9)	64.0 (10.3)	78.9 (11.8)	66.4 (12.4)
BMI, kg/m^2^ (SD)	20.5 (3.5)	20.9 (3.6)	23.0 (3.4)	22.5 (3.2)	24.1 (3.0)	23.3 (4.1)
***Outcome***						
sACE2 (NPX)	3.4 (0.4)	3.2 (0.3)	3.6 (0.5)	3.3 (0.4)	3.6 (0.4)	3.3 (0.4)

*BMI* body mass index, *sACE2* serum angiotensin-converting enzyme 2, *NPX* Normalized Protein eXpression

There was similar and low sACE2 in both sexes up to age 12. sACE2 increased more in boys with growth, so men from age 15 had higher sACE2 than women (Fig. 1). Thus, sACE2 is low in children and increases more in boys than girls, resulting in sex differences in adolescence/young adulthood.

**Fig. 1:**
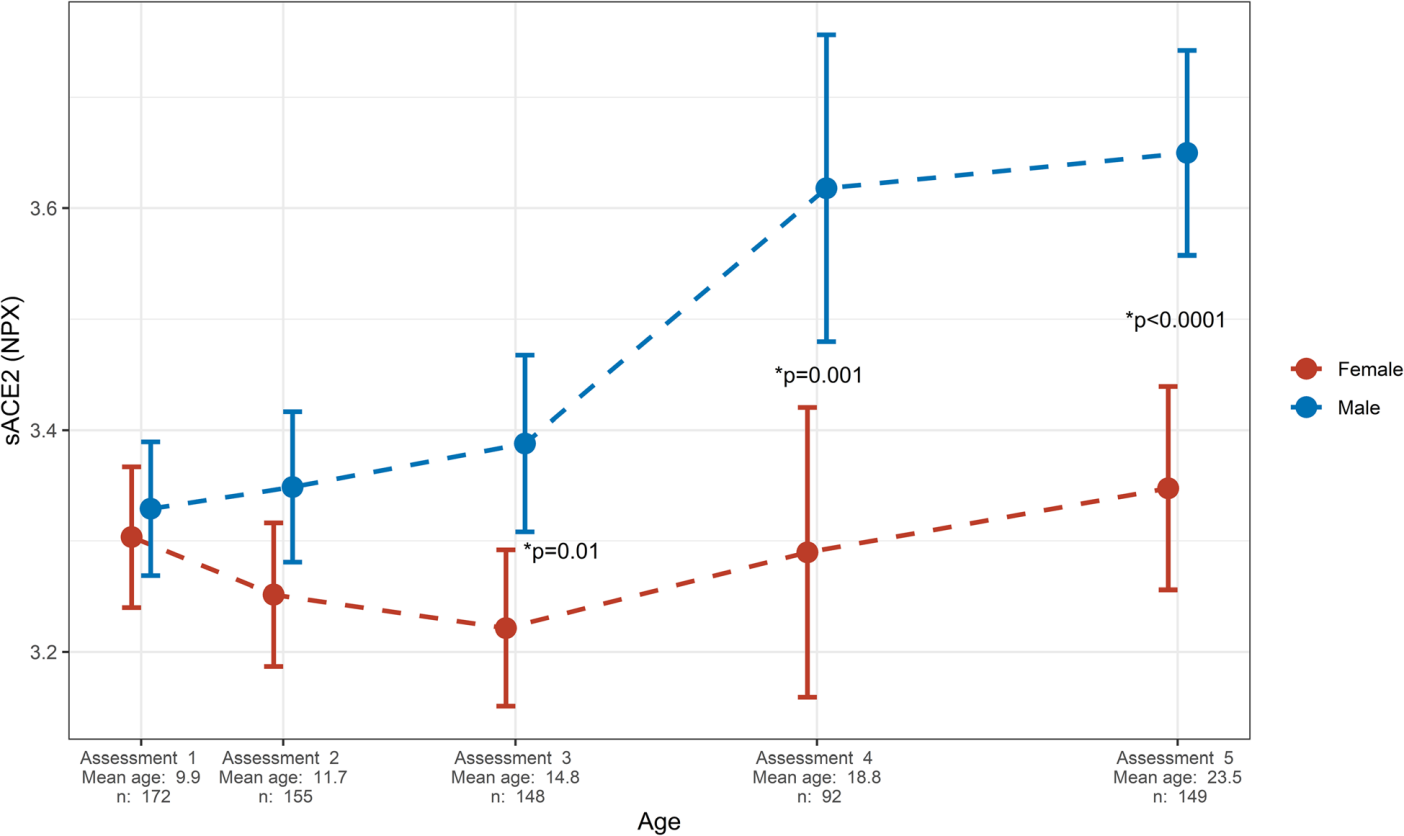
Sex-specific levels of sACE2 in relation to age. Data is presented as Normalized Protein eXpression (NPX) values, which is an arbitrary unit on a log2 scale, as mean with error bars representing 95% confidence intervals

SARS-CoV and SARS-CoV-2 share many features, including that the spike proteins, which bind mACE2, have almost identical 3-D structure in the receptor-binding domain [1]. Animal and in vitro studies on SARS-CoV point to the importance of mACE2, and ADAM-17-mediated mACE2 shedding, for the development of severe ALI [1, 2]. For example, overexpression of human ACE2 increases disease severity in SARS-CoV-infected mice [1]. Also, ADAM-17 silencing decreases host cell entry of SARS-CoV [2]. The spike protein of the coronavirus HNL63CoV, which results only in common cold, does not induce mACE2 shedding [2]. High mACE2 and/or high ADAM-17 activity may therefore be risk factors for severe COVID-19 [1, 2]. Since high sACE2 could indicate high mACE2 and/or high ADAM-17 activity, sACE2 may be a marker of both susceptibility and severity of COVID-19.

The longitudinal study design is a strength of the present study. Study limitations include only following the subject into young adulthood, description of normal biology only, and the inability to, in serum, analyze mACE2 and cell ADAM-17 activity. However, the cleavage and the release of mACE2 due to ADAM-17 activity have already been well characterized [2, 3].

In conclusion, this study shows that subjects with higher risk for severe COVID-19 [4] had higher sACE2 (adults>children and men>women). We suggest that further studies evaluate if high sACE2 is a risk factor for severe COVID-19 and to what extent sACE2 is related to increased ADAM-17 activity and mACE2 shedding.

## Data Availability

All data generated or analyzed during this study are included in this published article. The corresponding author (PS) can be contacted for more information.

## Abbreviations

mACE2: Membrane-bound angiotensin-converting enzyme 2
sACE2: Soluble angiotensin-converting enzyme 2
COVID-19: Coronavirus disease 2019
SARS-CoV: Severe acute respiratory syndrome coronavirus
ADAM-17: A disintegrin and metalloproteinase-17
ALI: Acute lung injury
RAS: Renin-angiotensin-system
POP study: Pediatric osteoporosis prevention study
NPX: Normalized Protein eXpression
